# Generating Intervention Concepts for Reducing Adolescent Relationship Abuse Inequities Among Sexual and Gender Minority Youth: Protocol for a Web-Based, Longitudinal, Human-Centered Design Study

**DOI:** 10.2196/26554

**Published:** 2021-04-12

**Authors:** Robert W S Coulter, Shannon Mitchell, Kelly Prangley, Seth Smallwood, Leyna Bonanno, Elizabeth N Foster, Abby Wilson, Elizabeth Miller, Carla D Chugani

**Affiliations:** 1 Department of Behavioral and Community Health Sciences Graduate School of Public Health University of Pittsburgh Pittsburgh, PA United States; 2 Department of Pediatrics University of Pittsburgh School of Medicine Pittsburgh, PA United States; 3 Division of Adolescent and Young Adult Medicine UPMC Children's Hospital of Pittsburgh Pittsburgh, PA United States; 4 LUMA Institute Pittsburgh, PA United States

**Keywords:** sexual and gender minorities, adolescent, psychosocial intervention, internet-based intervention, intimate partner violence

## Abstract

**Background:**

Sexual and gender minority youth (SGMY; eg, lesbian, gay, bisexual, and transgender youth) are at greater risk than their cisgender heterosexual peers for adolescent relationship abuse (ARA; physical, sexual, or psychological abuse in a romantic relationship). However, there is a dearth of efficacious interventions for reducing ARA among SGMY. To address this intervention gap, we designed a novel web-based methodology leveraging the field of human-centered design to generate multiple ARA intervention concepts with SGMY.

**Objective:**

This paper aims to describe study procedures for a pilot study to rigorously test the feasibility, acceptability, and appropriateness of using web-based human-centered design methods with SGMY to create novel, stakeholder-driven ARA intervention concepts.

**Methods:**

We are conducting a longitudinal, web-based human-centered design study with 45-60 SGMY (aged between 14 and 18 years) recruited via social media from across the United States. Using MURAL (a collaborative, visual web-based workspace) and Zoom (a videoconferencing platform), the SGMY will participate in four group-based sessions (1.5 hours each). In session 1, the SGMY will use rose-thorn-bud to individually document their ideas about healthy and unhealthy relationship characteristics and then use affinity clustering as a group to categorize their self-reported ideas based on similarities and differences. In session 2, the SGMY will use rose-thorn-bud to individually critique a universal evidence-based intervention to reduce ARA and affinity clustering to aggregate their ideas as a group. In session 3, the SGMY will use a creative matrix to generate intervention ideas for reducing ARA among them and force-rank the intervention ideas based on their potential ease of implementation and potential impact using an importance-difficulty matrix. In session 4, the SGMY will generate and refine intervention concepts (from session 3 ideations) to reduce ARA using round robin (for rapid iteration) and concept poster (for fleshing out ideas more fully). We will use content analyses to document the intervention concepts. In a follow-up survey, the SGMY will complete validated measures about the feasibility, acceptability, and appropriateness of the web-based human-centered design methods (a priori benchmarks for success: means >3.75 on each 5-point scale).

**Results:**

This study was funded in February 2020. Data collection began in August 2020 and will be completed by April 2021.

**Conclusions:**

Through rigorous testing of the feasibility of our web-based human-centered design methodology, our study may help demonstrate the use of human-centered design methods to engage harder-to-reach stakeholders and actively involve them in the co-creation of relevant interventions. Successful completion of this project also has the potential to catalyze intervention research to address ARA inequities for SGMY. Finally, our approach may be transferable to other populations and health topics, thereby advancing prevention science and health equity.

**International Registered Report Identifier (IRRID):**

DERR1-10.2196/26554

## Introduction

### Background

Sexual and gender minority youth (SGMY; eg, lesbian, gay, bisexual, and transgender youth) are at greater risk than their cisgender heterosexual peers for experiencing adolescent relationship abuse (ARA; ie, physical, sexual, or psychological abuse in a romantic relationship) [1-4]. According to a recent nationally representative sample of high school youth in the United States, 13% of sexual minority youth and only 7% of heterosexual youth reported physical ARA in the past year [5]. Even greater disparities are present for sexual ARA, with a prevalence of 16% among sexual minority youth and only 7% among heterosexual youth [5]. Gender minority youth are also at greater risk for ARA than their cisgender peers [6]. SGMY ARA inequities are problematic because ARA is associated with many poor health outcomes later in life, such as mental health disorders and HIV [7,8]. Thus, prevention efforts for SGMY may mitigate health inequities more broadly.

Despite researchers and national agencies calling for interventions to reduce ARA among SGMY [9-11], there exist few evidence-based interventions addressing this public health inequity [9,12]. While there are several efficacious interventions for reducing ARA for the entire adolescent population [13], a 2019 systematic review revealed that there were no evidence-based ARA interventions specifically for SGMY at that time [12]. More recently, one study examined the efficacy of a universal intervention for reducing ARA among sexual minority youth; for sexual minority youth, this intervention reduced stalking victimization but not sexual violence, sexual harassment, and physical dating violence victimization [14]. More research is needed to address the lack of evidence-based interventions to prevent and reduce ARA in SGMY.

One innovative method for stimulating new stakeholder-driven intervention ideas to catalyze ARA research among SGMY is to leverage the field of human-centered design [15]. Human-centered design is a discipline focused on improving existing or developing new products, services, or experiences by involving the perspectives of the target population at every possible stage [16-27]. Human-centered design methods often incorporate multiple ways of soliciting user and other stakeholder input, including through observation and dialog, cooperative design activities, and the shared creation of meaning by collaboratively synthesizing, critiquing, and ranking self-reported data and observations. In a practical sense, this often means that individuals involved in human-centered design processes create their own artifacts, including assembling, disassembling, and reassembling qualitative data points written on Post-it notes or a digital analog.

When bringing stakeholders together to design interventions, human-centered design generally harnesses the strengths and limits the weaknesses of more traditional approaches (eg, focus groups). For example, focus groups traditionally use group-based discussions and interviews generally work with participants one-on-one. Human-centered design methods, however, often combine tasks to be completed in groups with tasks to be completed as individuals, thereby harnessing the strengths of focus groups and interviews [28,29]. Focus groups are also prone to groupthink (conformity of individuals working in groups, despite their individual differences, which can lead to inaccurate results or poor decision making [30]) that may inadvertently reinforce social hierarchies that silence certain people (eg, marginalized or shy people) [31-34]. In contrast, many human-centered design techniques require each participant to brainstorm independently and record all their ideas in written format (before sharing output with other group participants), making data collection more comprehensive and equitable [28,35]. In addition, focus groups are prone to social desirability bias because the moderator has an active role in guiding and influencing the discussion [32-34,36]. In human-centered design, facilitators have a less subjective role as they usually only lead participants through task instructions without any probing [28]. Finally, conventional methods (eg, focus groups or expert panels) for bringing stakeholders to translate research findings into intervention concepts are often challenging and time-intensive [37-41]. To overcome these barriers, human-centered design uses structured activities that are time-limited, engaging, and accessible to laypersons, including youth [16-27]. Overall, human-centered design activities can be applied as a novel form of stakeholder-engaged research to rapidly generate and iterate intervention concepts to reduce emergent public health problems.

To date, health research that uses human-centered design methods has been predominantly conducted with stakeholders in-person [42], but most in-person research activities have been impeded by the COVID-19 pandemic. Although, the pandemic has presented many barriers for safely conducting in-person research, it has simultaneously catalyzed the use of web-based technologies for interacting and collaborating. Young people have especially become accustomed to using web-based technologies (eg, Zoom, a videoconferencing platform) because many schools have transitioned to remote learning. Therefore, youth are uniquely poised to use web-based human-centered design methods. Previous research has shown that youth can feasibly engage in web-based research as well as in-person human-centered design activities [43-47]. However, to our knowledge no study has explicitly tested whether human-centered design methods are feasible, acceptable, and appropriate for engaging with youth in a fully web-based environment. A study that rigorously pilot tests such methods, by setting a priori benchmarks, can help inform the public health field about the utility of conducting these methods on the web and further demonstrate and codify the use of web-based human-centered design as a method for stakeholder-engaged research. 

### Study Aims

This paper describes a protocol for conducting web-based human-centered design sessions with SGMY to create novel intervention ideas for addressing ARA. The specific aims of this study are as follows:

1. Test the feasibility, acceptability, and appropriateness of conducting web-based human-centered design sessions with SGMY (primary study aim).

2. Elucidate the beliefs of SGMY about healthy and unhealthy characteristics of intimate relationships.

3. Elicit feedback from SGMY about the School Health Center Healthy Adolescent Relationships Program (SHARP), which is a universal evidence-based intervention for reducing ARA [48,49], and about adapted SHARP materials that are tailored to SGMY [50,51].

4. Brainstorm intervention ideas for reducing ARA inequities for SGMY and force-rank the intervention ideas based on their potential ease of implementation and potential impact.

5. Generate, iterate, vote on, and refine the intervention concepts to reduce ARA inequities for SGMY.

## Methods

### Overview

We followed both the Standards for Reporting Qualitative Research [52] and the Strengthening the Reporting of Observational Studies in Epidemiology statement [53] to craft our study, present our methods, and report our results. We used these two guidelines because there are no formal reporting requirements for human-centered design studies.

### Study Design

**Figure 1:**
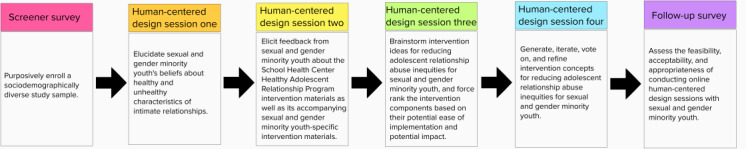
Study flow.

We are conducting a web-based, longitudinal human-centered design study to engage with 45 to 60 SGMY participants in small groups to generate intervention concepts for reducing SGMY inequities in ARA. We conducted longitudinal sessions in multiple cohorts of 8 to 15 participants each. Participants completed a web-based screener, four web-based, group-based human-centered design sessions, and a web-based follow-up survey (Figure 1). This study is primarily funded by the National Center for Advancing Translational Sciences at the National Institutes of Health (UL1TR001857).

### Study Population

We aim to enroll 45 to 60 sexual and gender minority high school students recruited via social media advertisements. Eligible youth are aged 14 years to 18 years, live in the United States, identify as sexual and/or gender minorities, and have internet, video camera, audio, and microphone access to attend the virtual sessions.

### Recruitment

Participants are conveniently sampled and recruited throughout the United States using web-based advertisements posted on two social media platforms, Facebook and Instagram, using an approach similar to that in our previous research [54]. This approach allows SGMY from multiple geographic locations (eg, rural and urban areas and East and West) to enroll in the study without overextending our resources. Facebook is an appropriate recruitment platform because it is highly used by adolescents, approximately 71% of teens use Facebook [55]. Similarly, Instagram is used by approximately 72% of teens in the United States, with the majority using the site daily [56]. We created multiple photo and video advertisements for these sites, including those depicting youth with diverse gender expressions, race, and ethnicities.

### Screener Survey

Upon clicking the advertisements, potential participants are redirected to a brief web-based self-reported screener survey administered via Research Electronic Data Capture (REDCap), a free and secure Health Insurance Portability and Accountability Act–compliant system for managing web-based surveys and databases. Following a brief description of the study, the screener included questions about potential participants’ age, race, ethnicity, sexual identity, sex assigned at birth, gender (including transgender status), high school name, city and state of their high school, computer access, camera access, audio access, microphone access, and contact information. All participants received the ARA and SGMY resource lists after the screener.

### Purposive Sampling

After potential participants completed the screener survey, a research assistant will assess their eligibility. The research assistant will send a sociodemographically diverse group of eligible youth a web-based consent form, which must be completed to participate.

### Consent Process

Potential participants are sent a link to a web-based consent form administered via DocuSign, a website that allows participants to safely and securely use a virtual signature. Youth in this study will consent for themselves because we obtained a waiver of parental consent. Our study is no more than minimal risk and requiring SGMY to obtain parental permission could *out* them as SGMY to their parents or guardians, which may put them at an increased risk of experiencing abuse. The consent form described all essential components of the study, including (but not limited to) the study purpose, the study background, study risks and benefits, privacy and confidentiality, participant payments, and voluntary nature of the study.

Once the consent form is virtually signed, the research assistant receives a PDF version of the signed form and emails or texts participants a link to the Zoom videoconference meeting, where the web-based human-centered design sessions are conducted. The message includes instructions on how to best prepare for the session and how to access MURAL, the web-based collaborative workspace used during our web-based sessions. The participants are sent reminders 2-3 times before each session.

In addition to the web-based consent form, participants provide verbal consent at the beginning of each web-based human-centered design session. A research assistant reads aloud a verbal consent script, asks if there are any questions, and then participants provide their consent by using Zoom’s *thumbs up* or via Zoom’s chat feature. In addition, before completing the follow-up survey, a brief consent script is provided to participants and participants voluntarily consented to taking the survey using a *click-to-consent* procedure.

### Human-Centered Design Activities

We use MURAL and Zoom to conduct the web-based human-centered design activities. With each cohort of SGMY, we will conduct 4 sessions, each lasting 1.5 hours in length. All the sessions will be conducted in English. We will audio-record each session and take pictures of each session’s activity results. We only record the participants’ voices and the resultant data (not images of participants). The participants will receive a US $25 incentive for each session. Participants do not have to attend all sessions and can begin at any time, although we encourage attendance at all sessions because output from some sessions are then used as inputs in subsequent sessions.

Each session begins with an introduction to Zoom, an icebreaker, and (in the first 2 sessions) a short lesson on the topic at hand (ie, ARA in SGMY). Next, the participants are randomly assigned to different Zoom breakout rooms, with each room composed of 2 to 5 participants with 1 or 2 facilitators each. In these small groups, the facilitators introduce participants to MURAL and guide the participants through a series of human-centered design activities. Each session’s human-centered design activities are outlined and briefly described in Table 1 and described in detail in the sections below. At the end of each meeting, all participants are brought back together and report on the ideas generated during the human-centered design activities.

**Table 1:** Human-centered design activities by session.

Session and activity		Purpose
**Session 1**		
	Rose, thorn, bud	To have participants individually brainstorm healthy, unhealthy, and questionable aspects of intimate relationships for SGMY^a^
	Affinity clustering	To have participants discuss all the healthy, unhealthy, and questionable aspects of intimate relationships they generated, and to have participants group similar ideas together
**Session 2**		
	Rose, thorn, bud	To have participants provide feedback on the original SHARP^b^ materials or the adapted SGMY-specific SHARP materials
	Affinity clustering	To have participants discuss all their feedback on the SHARP-related materials and to have participants group similar ideas together
**Session 3**		
	Creative matrix	To have participants brainstorm intervention ideas at each level of the social-ecological model for reducing SGMY inequities in adolescent relationship abuse
	Impact-difficulty matrix	To have participants plot their self-generated intervention ideas based on their potential ease of implementation and potential impact, thereby prioritizing the intervention ideas with the lowest potential resource expenditure and greatest potential impact
**Session 4**		
	Round robin	To have participants evolve their intervention ideas into fuller intervention concepts using quick drafting and iteration via group authorship
	Visualize the vote	To quickly poll SGMY’s preferences and opinions about two of their favorite intervention concepts
	Concept poster	To have participants work together to refine intervention concepts by illustrating and describing its essential elements

^a^SGMY: sexual and gender minority youth.

^b^SHARP: School Health Center Healthy Adolescent Relationships Program.

#### Session 1

**Figure 2:**
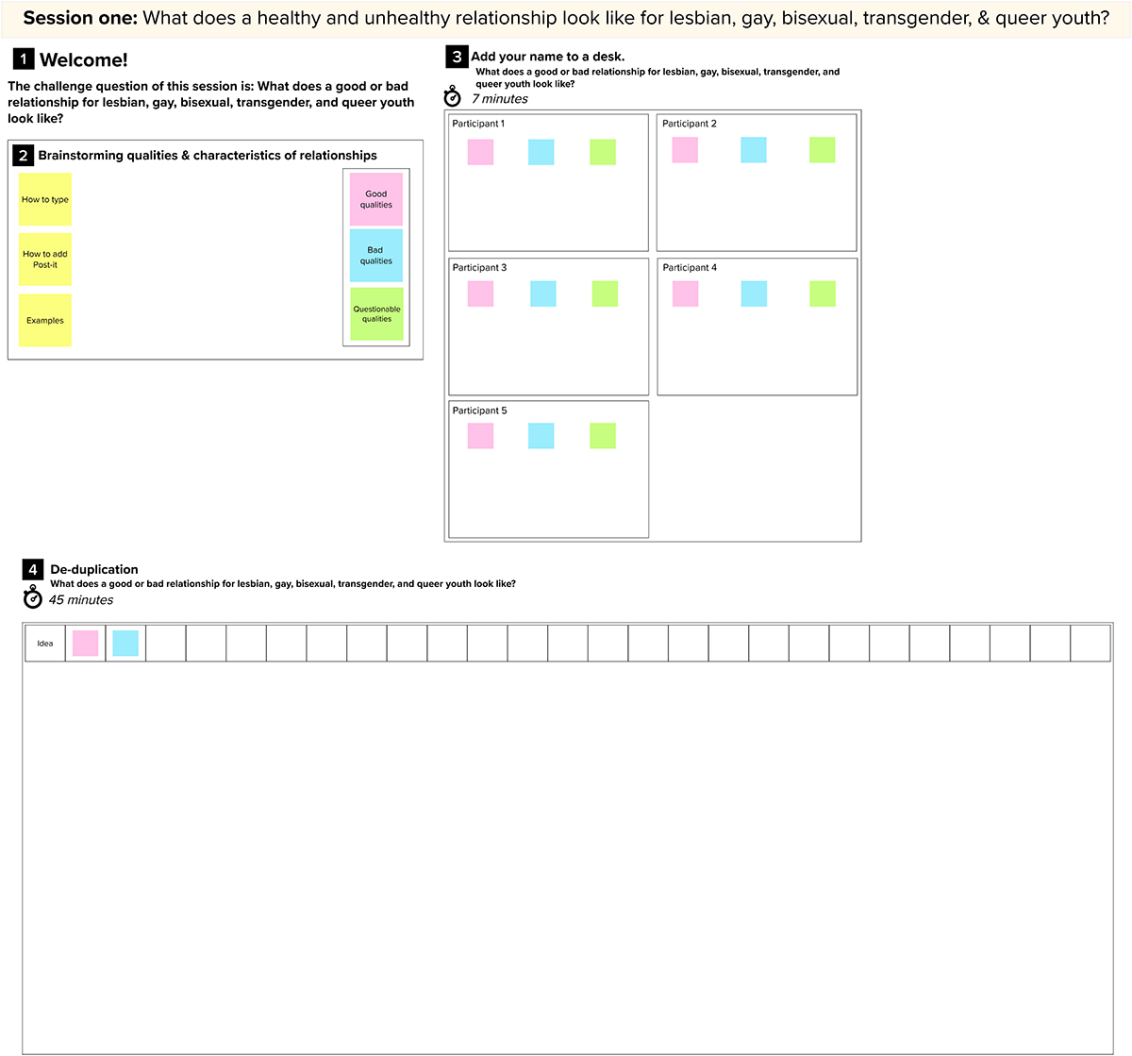
MURAL workspace setup for session 1.

The purpose of our first session is to elucidate what SGMY believe are healthy and unhealthy characteristics of intimate relationships (study aim 2). We will accomplish this by using 2 human-centered design techniques: rose-thorn-bud and affinity clustering activities. Both activities are set up in a single MURAL workspace (Figure 2).

Rose-thorn-bud is a technique in which participants identify different aspects of a concept [20,21], in this case, healthy intimate relationships. Each participant is assigned a virtual desk (Figure 2) with 3 different colored sticky notes: pink, blue, and green. The facilitator explains the color-coding system to the participants. Participants are to type aspects of healthy relationships on pink sticky notes (or *roses*); they are then instructed to type components or aspects of unhealthy relationships on blue sticky notes (or *thorns*); and green sticky notes (or *buds*) are used to note aspects of relationships that are uncertain qualities with potential to be healthy and/or unhealthy. The facilitator shows participants how to create additional sticky notes and instructs the participants to write one idea per sticky note, generating as many sticky notes as possible. For 7 minutes, participants work independently to complete their sticky notes.

Affinity clustering is used after completing the rose-thorn-bud activity to sort sticky notes according to their similarities and differences [20,21]. The facilitator asks a participant to read and explain one of their sticky notes (of any color) and the facilitator places it along the horizontal axis. The facilitator then asks another participant to describe one of their sticky notes and identify whether it is a similar or unique idea compared with the previously placed sticky note. Once determined, the facilitator then places this sticky note on the workspace below the previous sticky note if similar, or next to the previous sticky note if unique. This process is repeated until all sticky notes are in the workspace and are grouped according to the participants’ satisfaction. It is important to note that during affinity clustering, the facilitator encourages participants to align ideas based on its *content*, as opposed to the *color* of the sticky note. This type of clustering allows participants (as well as analysts who later view or interpret the data) to obtain 2 layers of meaning: the overall concepts of the groupings as well as what color sticky notes comprise those groupings. Overall, session 1 yields a visual representation of the SGMY’s mental models of intimate relationships.

#### Session 2

**Figure 3:**
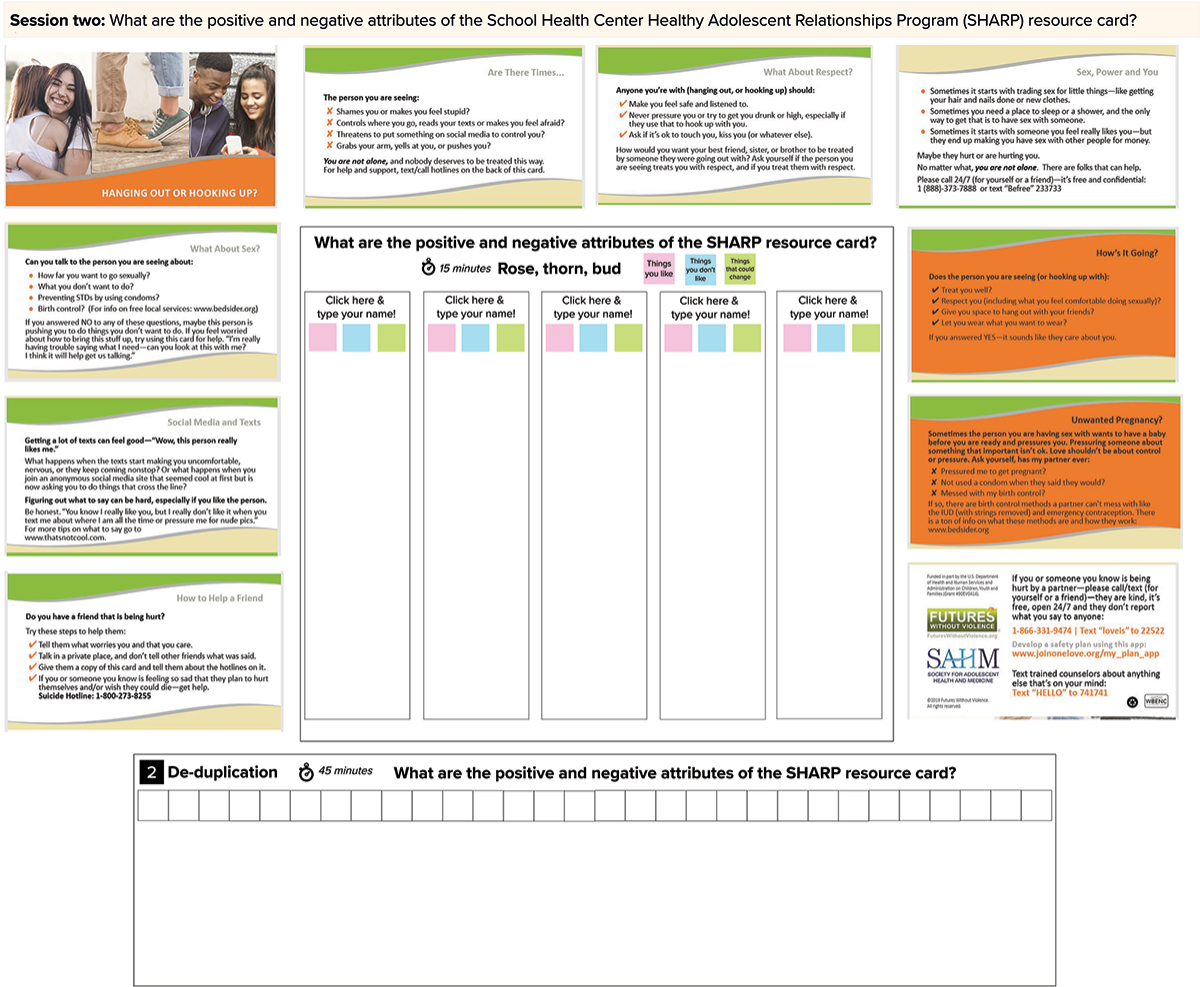
MURAL Workspace Setup for Session 2 on Original School Health Center Healthy Adolescent Relationships Program (SHARP) Intervention.

**Figure 4:**
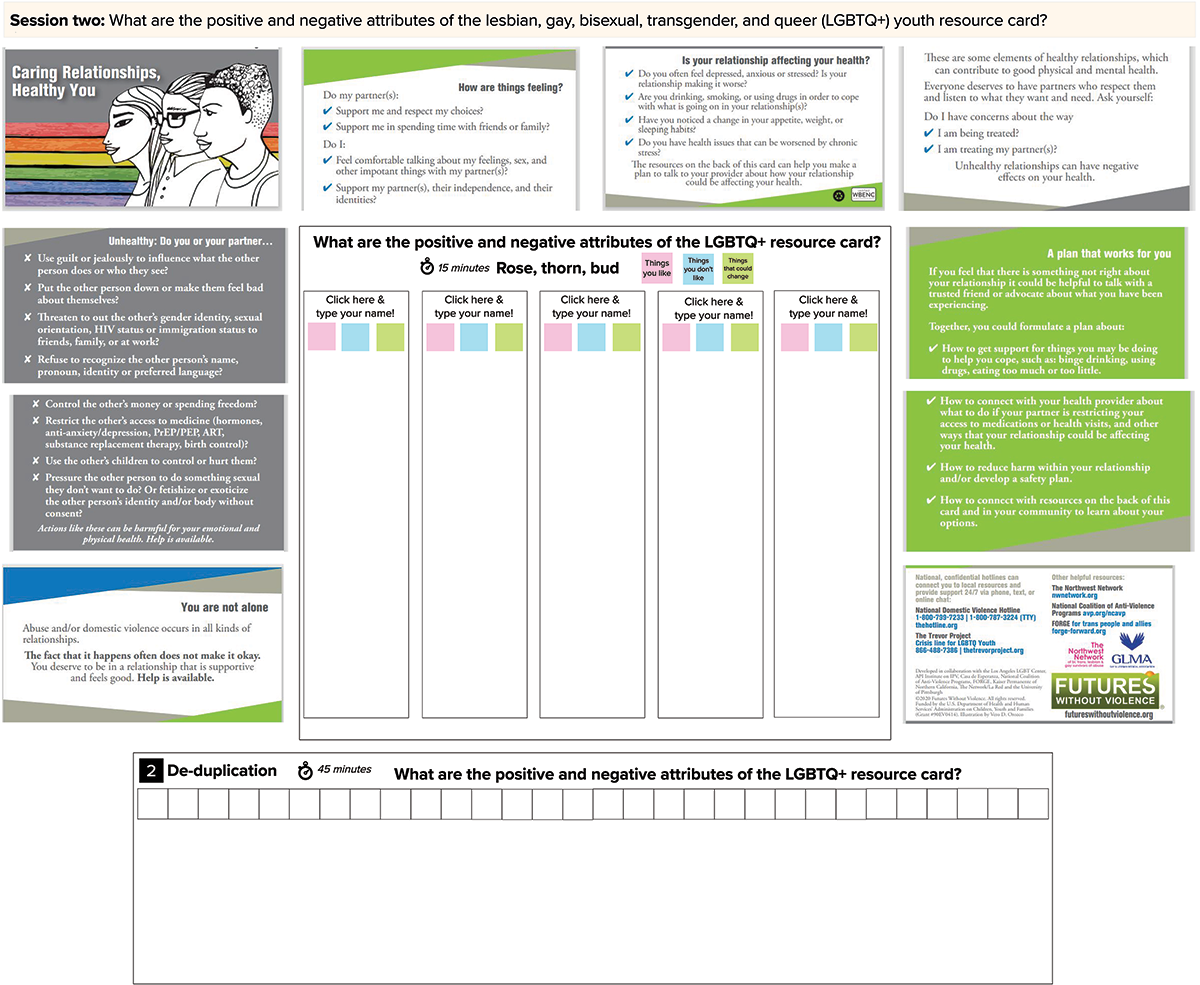
MURAL Workspace Setup for Session 2 on Adapted School Health Center Healthy Adolescent Relationships Program (SHARP) Materials Tailored Specifically for Sexual Minority Youth.

The purpose of session 2 is to acquire feedback from SGMY about the SHARP intervention materials as well as the adapted SGMY-specific SHARP materials (study aim 3) [48-51]. SHARP is a universal (not SGMY-specific) provider-based intervention implemented in routine school-based health center visits [48]. During clinic visits, SHARP providers introduce a palm-size brochure that contains information about healthy relationships and ARA resources, conduct ARA assessments, make referrals to ARA services, if necessary, and discuss healthy and unhealthy relationships with their patients. All SHARP materials were developed with input from numerous stakeholders, including clinicians, advocates, researchers, and youth. Compared with usual care, SHARP improves adolescents’ recognition of ARA, knowledge of ARA resources, and self-efficacy in using ARA harm reduction behaviors [48]. After the universal SHARP materials were created, investigators and stakeholders (including youth) generated SGMY-specific brochure materials tailored to SGMY [50,51]. We selected the SHARP intervention because (1) it is an illustrative example of an efficacious ARA prevention intervention and study participants may not be familiar with ARA interventions and (2) SGMY can provide feedback about the evidence-based intervention, which might confirm the applicability of the materials to SGMY in 2020 or offer ways to improve the brochure for current clinical practice or future research. We will have SGMY critique the SHARP brochure materials using two human-centered design techniques: rose-thorn-bud and affinity clustering activities. Both of these are set up in a single MURAL workspace (Figures 3 and 4).

Rose-thorn-bud is a technique for having participants characterize different aspects of a concept (in this case, the SHARP intervention) as good, problematic, or having potential [20,21]. Each participant is assigned a virtual desk (Figures 3 and 4) with 3 different colored sticky notes: pink, blue, and green. The facilitator explained the color-coding system to the participants. Participants are asked to write the components and aspects of what they like about the SGMY intervention on pink sticky notes (or *roses*); they are then instructed to type components they dislike on blue sticky notes (or *thorns*); and green sticky notes (or *buds*) are used to note aspects that have the potential to be inclusive but could be improved. The facilitator shows participants how to create additional sticky notes and instructs the participants to write one idea per sticky note, generating as many sticky notes as possible. For 7 minutes, participants work independently to complete their sticky notes.

Affinity clustering is used after completing the rose-thorn-bud activity to sort items according to their similarities and differences [20,21]. The facilitator asks one participant to read and explain one of their sticky notes (of any color) and the facilitator places it along the horizontal axis. The facilitator then asks another participant to describe one of their sticky notes and identify whether it is a similar or unique idea compared with the previously placed sticky note. Once determined, the facilitator then places this sticky note on the workspace below the previous sticky note if similar, or next to the previous sticky note if unique. This process is repeated until all sticky notes are in the workspace and are grouped according to the participants’ satisfaction.

#### Session 3

**Figure 5:**
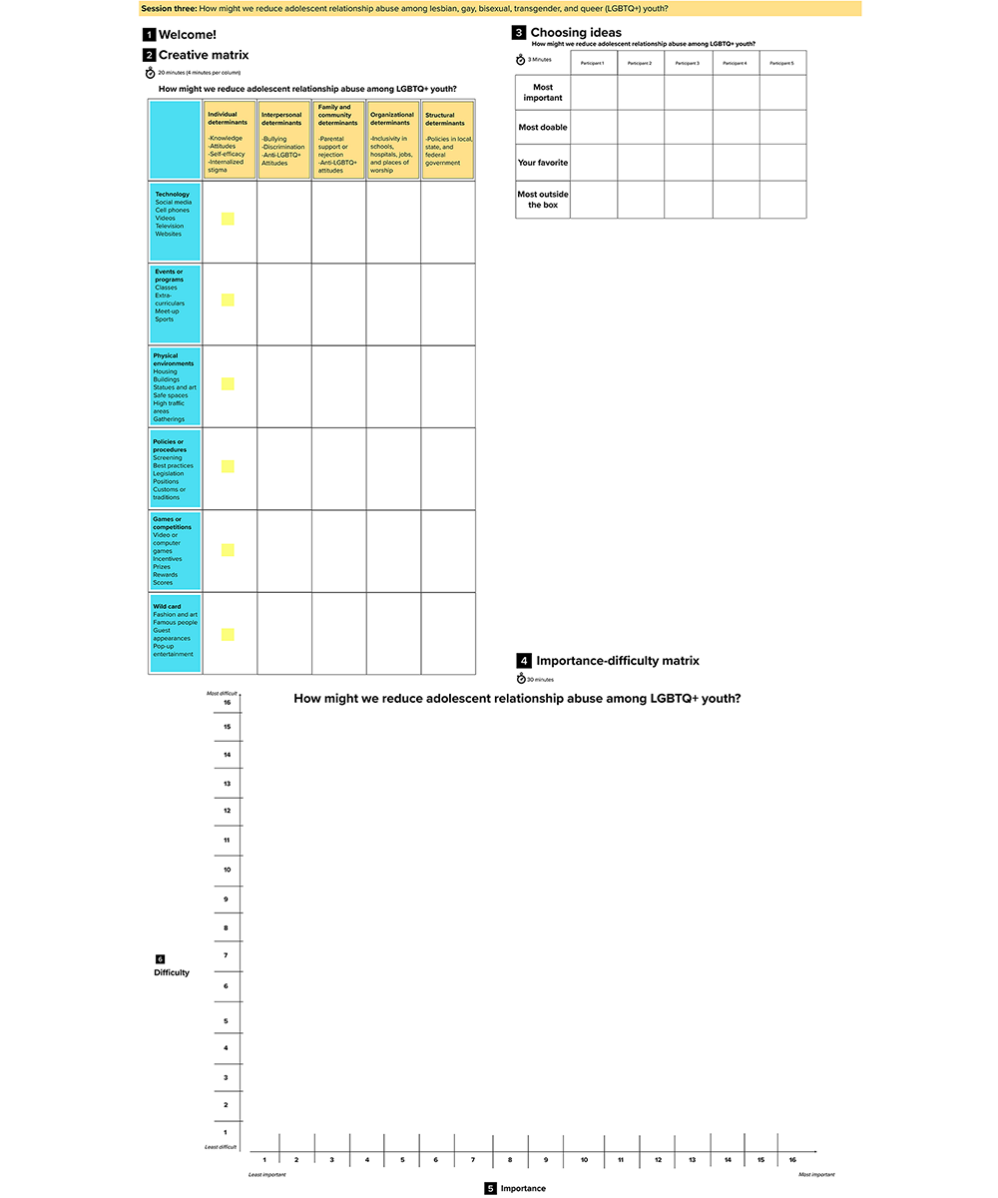
MURAL workspace setup for session 3.

The purpose of session 3 is to brainstorm novel intervention components to reduce ARA inequities for SGMY and force-rank the intervention components based on their potential ease of implementation and potential impact (study aim 4). We accomplish this by using 2 human-centered design strategies: a creative matrix and an importance-difficulty matrix. All of these are conducted in one MURAL workspace (Figure 5).

The creative matrix is used to brainstorm as many intervention concepts for reducing ARA among SGMY as possible by leveraging the power of constraints (column by column) and a combination of goals (column headings) and enablers (essentially categories of potential solutions) [16,18,20,21,23,26,27]. As shown in Figure 5, the column headers indicate different levels of the social-ecological model, that is, individual, interpersonal, family and community, structural, and organizational determinants, whereas the row headings indicate the modalities: technology, events or programs, physical environments, policies or procedures, games or competitions, and wild card (ie, any other ideas that do not fit with the labeled categories). By providing different levels of the social-ecological model, SGMY are primed to think about creating intervention components that address known risk factors (eg, minority stressors [57]) and protective factors that contribute to ARA inequities for SGMY [57-63]. The activity is completed in 1 column at a time, with 4 minutes per column. Participants are asked to develop ideas (one idea per sticky note) for each cell or box by working independently. Participants are working individually at this time, reducing groupthink (eg, influence of more vocal contributors). In this activity, participants are encouraged to generate as many ideas as possible within a short period. Emphasis is typically placed on quantity (over quality) to eliminate barriers to contribution, get participants comfortable with the activity, and give them the opportunity to share ideas they may have already had before being prompted with the matrix and create space for new ideas informed by exploration of each row and column combination.

After completing the creative matrix, participants engage in a structured activity to funnel the large number of ideas generated in the creative matrix into a manageable number of ideas to bring to the next activity. Facilitators ask participants to identify 4 sticky notes each: most important, most doable, their favorite, and the most out-of-the-box. These 4 criteria for *down-selection* were designed to ensure that ideas pursued in subsequent activities are sufficiently diverse. Each participant works independently for 3 minutes while they choose their ideas. The participants cannot choose the same ideas.

An importance-difficulty matrix [20-22,26] is then used to force-rank the ideas identified in the previous step. First, participants are asked to collaboratively rank the ideas that they chose on the level of importance along the x-axis. Importance here is defined as how important or impactful the participants think the idea is to reduce ARA for SGMY. Next, they are asked to rank these ideas on the level of difficulty along the y-axis while retaining the same ordered ranking of likely impact or importance. The purpose of this activity is to identify the intervention concepts that are the most important to the participants and, given the inevitable resource constraints, account for their potential difficulty to implement.

#### Session 4

**Figure 6:**
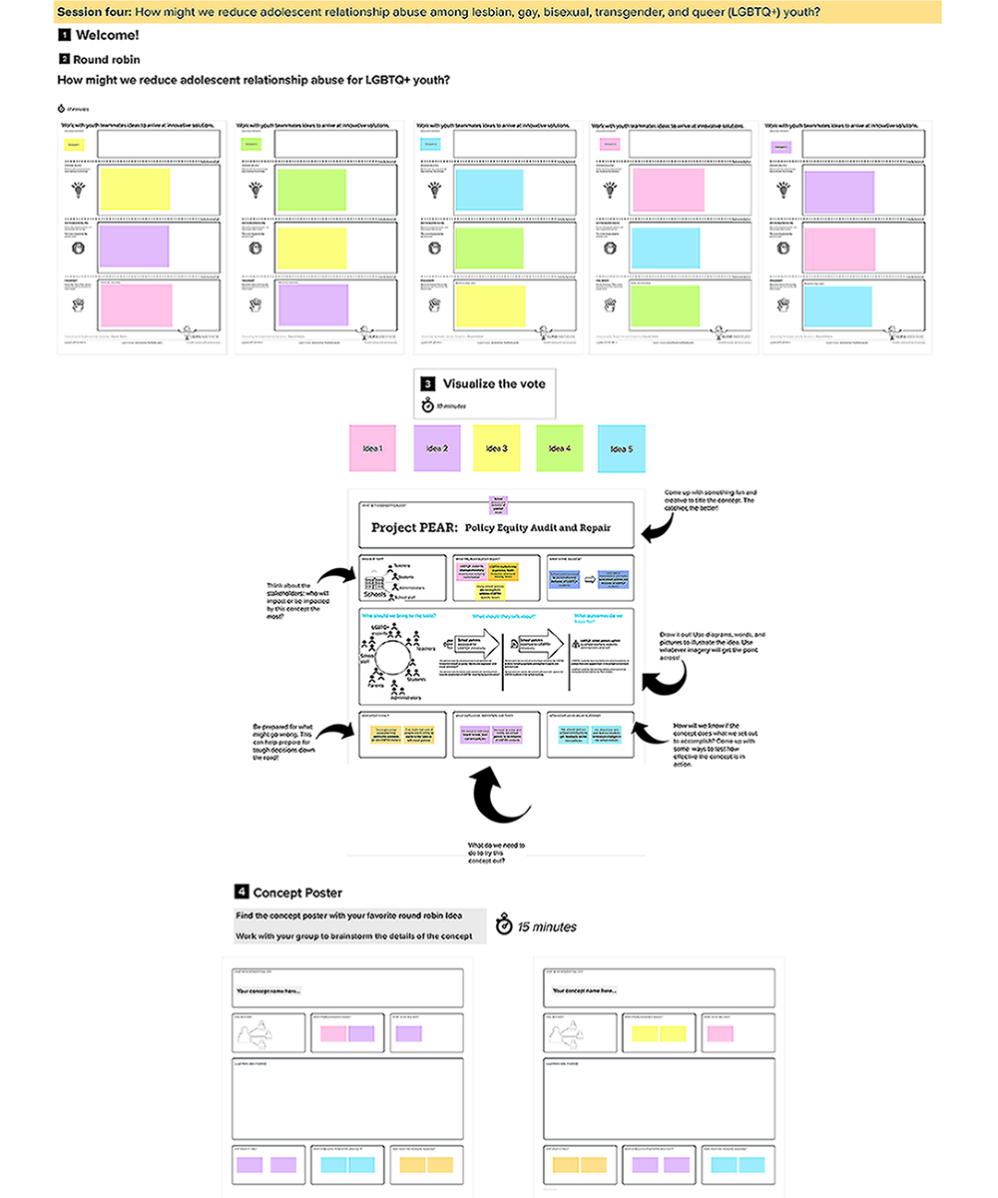
MURAL workspace setup for session 4.

The purpose of session 4 is to generate, iterate, vote on, and refine intervention concepts to reduce ARA inequities for SGMY (study aim 5). We accomplish this by using three human-centered design strategies: round robin, visualize the vote, and concept posters. All of these are set up in one MURAL workspace (Figure 6).

**Figure 7:**
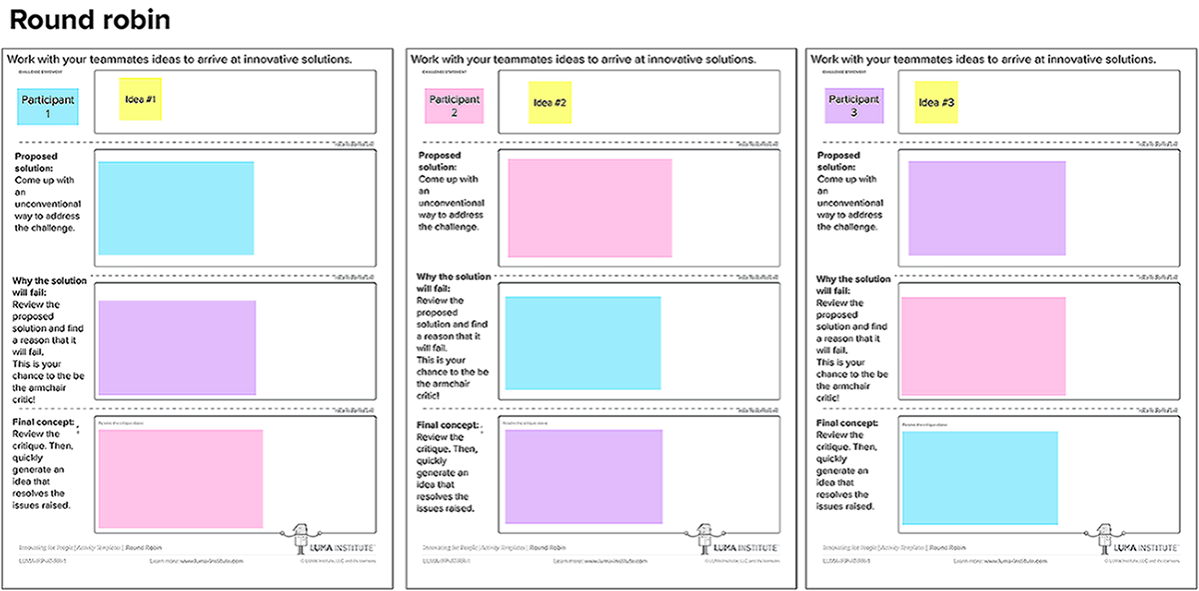
Example of one round robin setup in MURAL.

In total, 5 of the potentially most impactful and easiest to design and implement ideas generated from the importance-difficulty matrix in session 3 are carried over to session 4 for the round robin activity. Round robin is used to evolve ideas into fuller intervention concepts using drafting and iteration among participants [20,21,24,25]. Each desk has 4 sections: problem statement (idea from previous session), proposed solution, why the solution might fail, and the final concept. This structure was developed by the LUMA Institute (see Figure 7 for a reader-friendly version of the round robin structure) [20,21]. Each participant starts at their own desk (a specific rectangle in the MURAL workspace) with the initial intervention concept. For 3 minutes, participants are asked to propose a solution to their intervention concept. Then, participants rotate workspaces by moving to the space to their right (or, if at the last desk, moving to the first desk). Then, participants are asked to provide feedback on why the next intervention concept might fail for 5 minutes. Participants move to the desk to their right one final time and for 7 minutes generate a final concept based on the feedback provided by the previous participants.

Visualizing the vote is an activity that allows the facilitator to anonymously poll the participants and is a feature offered by MURAL [20,21]. After reading through the final round robin concepts, facilitators show participants where the voting ballots are and ask participants to vote for their 2 favorite concepts. Once this voting process is complete, 2 round robin concepts with the most votes overall are carried over to the final activity, the concept poster.

**Figure 8:**
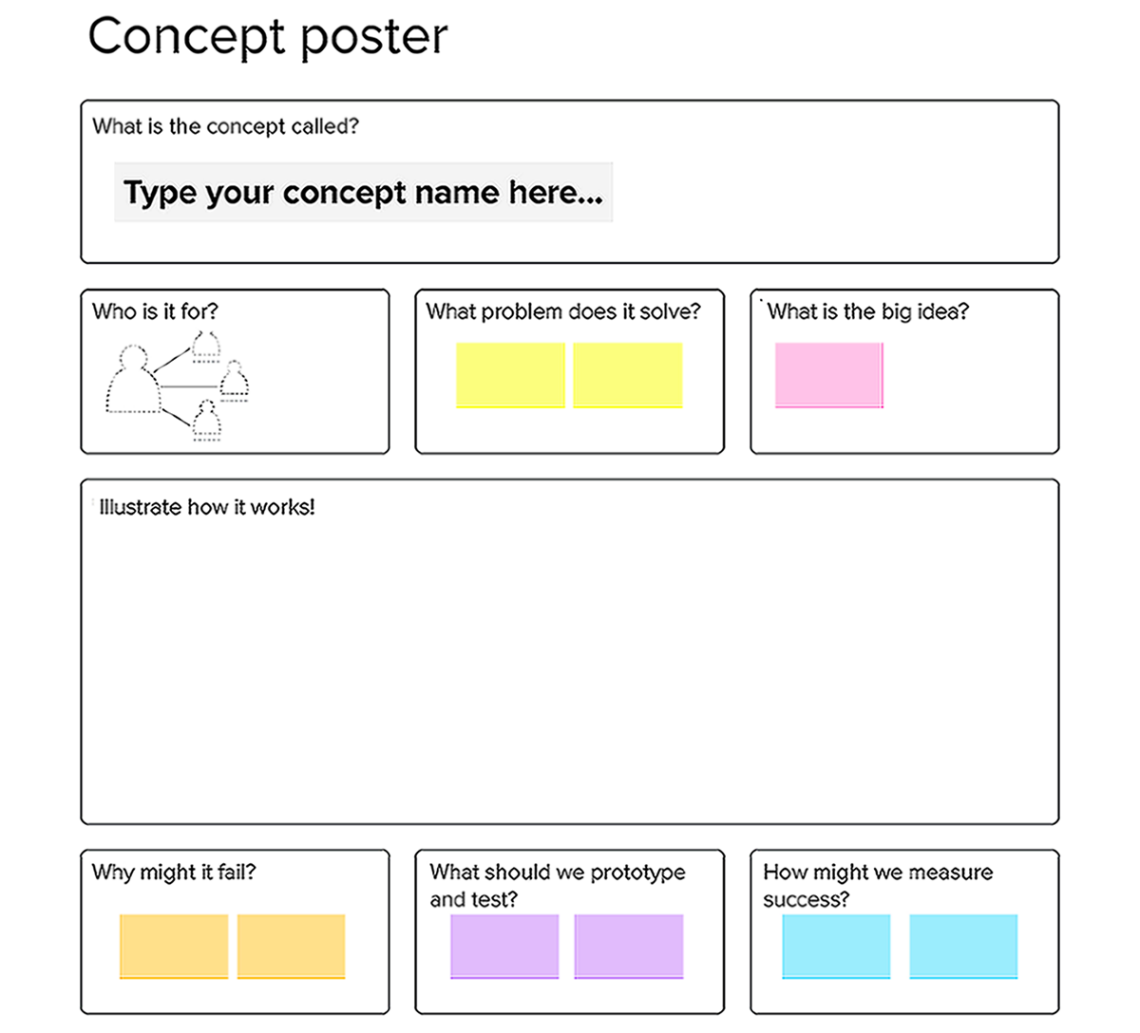
Example of one concept poster setup in MURAL.

The concept poster is used to illustrate and present the main points of the new intervention concepts [20,21]. The concept poster format, developed by the LUMA Institute [20,21], includes generating a creative title and identifying the following: the target population, the problem it solves, the big idea, how it works, why it might fail, what a prototype looks like, and how to measure success (see Figure 8 for a reader-friendly version of the concept poster activity). Facilitators first show the participants an example of a completed concept poster to illustrate the purpose of the concept poster. Participants are then invited to collaboratively develop 2 concept posters (allowing 15 minutes per poster). During this activity, facilitators answer participants’ questions and provide guidance on an as-needed basis.

#### Follow-Up Survey Data Collection

After session 4, participants are asked to complete self-administered follow-up surveys via REDCap. The follow-up surveys assess participants’ perceptions about the feasibility, appropriateness, and acceptability of the web-based human-centered design session, as well as participants’ qualitative feedback on session logistics. The follow-up survey is activated and sent within 1 week after session 4. Surveys remain open for up to 2 weeks. The follow-up survey contains 3 pages, with a mean of 10 items per page (SD 6; range 6-17). While completing each survey, participants were able to change their answers by clicking a *Back* button. An incentive of US $10 was given after completion of the follow-up survey.

#### Follow-Up Survey Measures

To accomplish aim 1, we assess participants’ perceptions of the feasibility, acceptability, and appropriateness of the web-based human-centered design sessions.

Feasibility is defined as the perception among participants that human-centered design sessions can be successfully implemented or carried out on the web [64]. Feasibility is measured using the Feasibility of Intervention Measure [64-66], which is a valid and internally consistent scale based on 4 positively worded items (eg, “The online sessions for this project were implementable”) that use a 5-point Likert scale ranging from *completely disagree* (1) to *completely agree* (5). We will create a mean across all items, with a higher mean score indicating greater feasibility.

Acceptability is defined as the perception among stakeholders that web-based human-centered design sessions are satisfactory or agreeable [66]. Acceptability is measured using the Acceptability of Intervention Measure [65-67], which is a valid and internally consistent scale based on 4 positively worded items (eg, “The online sessions for this project seemed doable”) that use a 5-point Likert scale ranging from *completely disagree* (1) to *completely agree* (5). We will create a mean across all items, with a higher mean score indicating greater acceptability.

Appropriateness is defined as the perception among participants that web-based human-centered design sessions are relevant, compatible, and suitable to address a specific issue [66]. Appropriateness is measured using the Intervention Appropriateness Measure [65], which is a valid and internally consistent scale based on 4 positively worded items (eg, “The online sessions for this project seemed applicable”) that use a 5-point Likert scale ranging from *completely disagree* (1) to *completely agree* (5). We will create a mean across all items, with a higher mean score indicating greater appropriateness.

In addition to our standardized measures, we ask participants exploratory open-ended questions including about what they liked most and liked the least about the web-based suggestions as well as ways to improve the sessions and retention. We also asked closed-ended questions about their opinions about session length and frequency.

### Data Analyses

For quantitative data analysis, we will use StataSE version 15 (StataCorp). For qualitative data analysis, we will transcribe, deidentify, and quality check all audio-recorded data from sessions 1 to 4 [68-71]. We will also export all session data from MURAL into text-based documents. We will then perform qualitative coding in Dedoose software [72], cross-referencing the audio transcriptions with PDF exports of the MURAL workspaces.

#### Sample Characteristics

Using data from the web-based screener survey, we will use frequencies and percentages to describe the demographics of our study sample.

#### Analyses for Study Aim 1

To assess the feasibility, acceptability, and appropriateness of conducting web-based human-centered design sessions with SGMY, we will calculate the means and 95% CIs for our primary outcome measures of feasibility, acceptability, and appropriateness. Our a priori benchmark for the success of meeting this hypothesis is obtaining a mean significantly higher than 3.75 (out of 5.00).

#### Analyses for Study Aims 2 to 5

To elucidate what SGMY believe are healthy and unhealthy characteristics of intimate relationships, we will use content analyses [73]. Using data from each session separately, we will code the artifact or audio-recording transcripts using an open-coding approach (ie, we will not create an a priori codebook, but will use *in vivo* coding instead) [68,69,74]. A total of 2 qualitative coders will independently read data from that session. Coders will convene to discuss preliminary findings and develop a draft codebook using inductive coding, allowing new codes to be included in the codebook as they emerge. Once the coders agree that all the major codes are identified, we will create a final codebook with definitions, rules, and examples for each code [70,71]. The 2 coders will then recode all data using the final codes. We will calculate the interrater reliability (ie, Kappa statistic) to examine code application between coders [75]. Coders will discuss any discrepancies until they reach an agreement; any disagreements will be discussed and resolved during research team meetings. We will use either a qualitative descriptive coding approach [76] (wherein we describe and count the number of code applications) or axial coding [77] (wherein we combine inductive codes into broader categories to define emerging patterns or themes). We will identify and interpret patterns in the data using thematic analysis, as informed by Braun and Clark [78].

### Sample Size and Power Calculation

We calculate statistical power based on our primary outcomes (study aim 1), per the best practices for feasibility studies [64,79-84]. Given a conservative sample size of 45 participants and 5% type I error rate, we have the ability to estimate a 95% CI margin of error ≤0.43 for our primary outcomes, which we derived from the largest upper 95% CI limit of the SD measures in previous studies that used the same outcome measures [16-27].

For our other study aims (study aims 2-5) about the outcomes of our human-centered design methods, the purpose is idea generation, not saturation [16-27]. Given our previous work implementing similar activities, a sample of 45 to 60 participants is sufficient to generate unique and useful ideas.

### Researcher Characteristics and Reflexivity

Our team represents a wide range of sexual identities (ie, gay, lesbian, queer, bisexual, and heterosexual) and gender identities (ie, nonbinary, cisgender women, and cisgender men). We also comprise a multidisciplinary multitiered team, representing the fields of public health, counseling, human-centered design, library science, medicine, medical anthropology, political science, psychology, rehabilitation science, social work, and many roles in academia, including undergraduate, masters, research assistants, assistant professors, and full professors. Our diverse representation of sexual and gender identities complements our range of multidisciplinary expertise and professional backgrounds in informing data collection and analysis through lived experience.

We acknowledge that there are several ways in which our backgrounds may have influenced our research. Primarily, our facilitators for the human-centered design sessions were sexual minorities and gender minorities. This could help put our SGMY participants at ease, especially in rare instances when the interviewer disclosed their sexual or gender minority identities to participants. Furthermore, the diversity and range of disciplines represented by our research team provide us with the ability to interpret our findings.

### Ethics Statement

All study procedures were approved by the Human Research Protection Office of the University of Pittsburgh. To protect participants from having to reveal their sexual or gender minority identities to their caregivers, thereby potentially putting them in harm’s way, we received a waiver of parental consent [85]. This allowed the participants to provide consent for themselves. To further protect participants, we asked SGMY (before and during each session) to find a quiet and private space where they could participate in activities with minimal distraction and interruption. We also allowed participants to use Zoom’s chat function if they are unable or feel uncomfortable speaking out loud. This study was also protected by a Certificate of Confidentiality from the National Institutes of Health. It is important to note that we never inquired about participants’ personal experiences with ARA. Nevertheless, we provided all people who completed the screener with the ARA and SGMY resource lists. In addition, the study participants received resource lists after each session.

## Results

**Figure 9:**
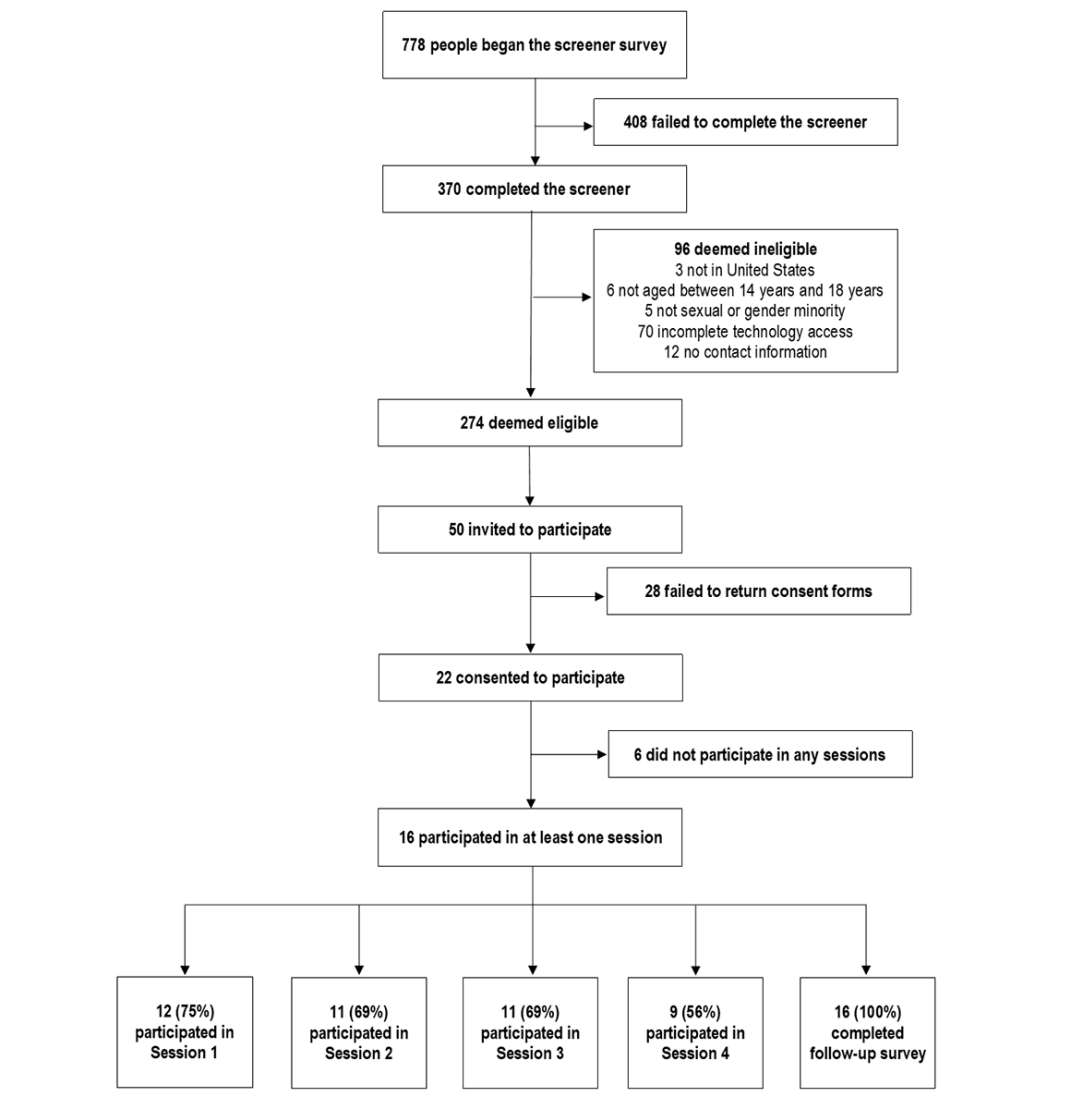
Flow diagram of the study as of December 2020.

This study was funded in February 2020. Data collection began in August 2020 and will be completed in April 2021. From August 2020 through December 2020, 778 individuals clicked the link to the screening questionnaire (Figure 9). In total, 370 individuals completed the screening questionnaire, of which 274 individuals met all eligibility criteria. A total of 50 participants were invited to participate, and 22 consented to participate. In total, 16 SGMY participated in at least one human-centered design session. All data collection will be completed by April 2021.

## Discussion

### Impact

This study protocol has several methodological innovations that can inform future public health research that aims to incorporate methods from the field of human-centered design. First, our method is an example of how human-centered design research can be performed on the web. This is important because it is a resource-friendly and accessible method for engaging diverse stakeholders from a wide geographic region. Moreover, the COVID-19 pandemic hindered our ability to engage safely with participants, and these web-based methods enable our research to continue despite current limitations on in-person research activities. Our study also demonstrates how to combine human-centered design, which is often quite flexible, into research processes, which tend to be much more structured. By rigorously testing the feasibility of such an approach, our study has the potential to demonstrate and codify the use of human-centered design as a novel stakeholder-engaged research method.

Our study has the potential to lead to many substantive innovations in the field of ARA interventions among SGMY. If successful, our study could yield several novel intervention concepts. Importantly, these interventions are directly derived from SGMY themselves, as opposed to researchers. By centering youth voices and opinions in this manner, the generated interventions may be highly acceptable and impactful, but that cannot be determined until the interventions are tested further. Nevertheless, given the great lack of interventions, our study can help catalyze the field of SGMY ARA intervention research.

### Limitations

Although there are many strengths to this study, it is not without limitations. Although participants in our sample are sociodemographically diverse, they are not necessarily representative. For example, SGMY without internet access are excluded from our study, and sessions were only conducted in English. In addition to youth, the inclusion of other stakeholders (eg, parents and school personnel) would likely have important insights for intervention concepts, but they are not included in this study. Despite our study yielding potentially new intervention concepts, this study will not produce complete interventions. Additional work from researchers, designers, and stakeholders will be necessary to develop and test the derived interventions. Finally, this is a pilot study testing the feasibility of our methods. We are not testing the effectiveness of our methods versus more traditional methods (eg, focus groups) for producing intervention concepts, which can be executed in future trials if our methodology proves to be feasible.

### Conclusions

Interventions to reduce ARA among SGMY are lacking. To address this gap, our study investigates the feasibility of a new method for generating new intervention concepts. This work has the potential to contribute to substantive health impacts as well as immediate methodological impact by integrating human-centered design methods into public health research.

## Abbreviations

ARA: adolescent relationship abuse
REDCap: Research Electronic Data Capture
SGMY: sexual and gender minority youth
SHARP: School Health Center Healthy Adolescent Relationships Program
